# MicroRNA-21 as a potential diagnostic biomarker for breast cancer patients: a pooled analysis of individual studies

**DOI:** 10.18632/oncotarget.9142

**Published:** 2016-05-02

**Authors:** Ying Gao, Qiliang Cai, Yubei Huang, Shu Li, Hongxi Yang, Li Sun, Kexin Chen, Yaogang Wang

**Affiliations:** ^1^ Department of Health Service Management, School of Public Health, Tianjin Medical University, Tianjin, 300070, China; ^2^ Department of Cancer Epidemiology and Biostatistics, Tianjin Medical University Cancer Institute and Hospital, Tianjin, 300060, China; ^3^ Department of Urology, The Second Hospital of Tianjin Medical University, Tianjin, 300211, China

**Keywords:** microRNA-21, breast cancer, diagnostic, biomarker, meta-analysis

## Abstract

MicroRNA-21 (miR-21) has been reported as the potential novel diagnostic biomarker for breast cancer in several studies, but their results were inconsistent. Therefore, we conducted a systematic analysis to evaluate the diagnostic value of miR-21 in detecting breast cancer. A comprehensive electronic and manual search was conducted for relevant literatures through several databases up to November 9, 2015. QUADAS-2 was used to assess the quality of the studies included in the study. All statistical analyses were performed using Meta-Disc 1.4 and Stata 12.0. Eleven studies with a total of 918 breast cancer patients and 613 controls were included. The pooled sensitivity, specificity, positive likelihood ratio (PLR), negative likelihood ratio (NLR), and diagnostic odds ratio (DOR) with their 95% confidence intervals (CIs) were 0.72 (95% CI: 0.69–0.75), 0.80 (95% CI: 0.77–0.83), 3.37 (95% CI: 2.24–5.07), 0.30 (95% CI: 0.19–0.50), and 11.79 (95% CI: 5.23–26.57), respectively. The area under the curve of SROC was 0.8517. In conclusion, our analyses suggested that miR-21 is a promising biomarker in diagnosing breast cancer. For clinical purpose, further large-scale studies are warranted to validate its clinical application.

## INTRODUCTION

Breast cancer is the most common cancer among women worldwide. Although breast cancer incidence rates still increases in many Western countries, mortality rates have been decreasing over the past two decades due to early detection and improved treatment [1]. The data from Surveillance, Epidemiology, and End Results Program (SEER) showed that the 5-year relative survival was 98.6% when diagnosed at localized stage as opposed to 23.3% when the disease at distant stage [2]. Thus, early detection and diagnosis has important clinical significances for breast cancer. The previous studies showed that the circulating tumor biomarkers such as carcinoembryonic antigen (CEA) and carbohydrate antigen 153 (CA153) are already applied in clinic, but these biomarkers are not useful to detect early breast cancer due to their low sensitivity and they have long been used as prognostic markers to monitor disease progression or recurrence [3–5].

After the first report of elevated circulating levels of microRNA-21 (miR-21) in patients with diffuse large B-cell lymphoma [6], circulating miRNAs with their stability feature have been postulated as novel biomarkers for cancer processes, such as liver cancer, ovarian cancer, breast cancer [7–9]. Several studies have reported miR-21 as the potential novel diagnostic biomarker for breast cancer, but their results were inconsistent. A recent study suggested that the circulating miR-21 could serve as a potential serum-based biomarker for breast cancer detection in Chinese population, with 80.0% sensitivity and 87.7% specificity [10]. Another study investigated the diagnostic accuracy of single miR-21 and reported a much lower sensitivity with 25.8% [11]. In the Asaga's study, significant up-regulation of miR-21 was detected, but it could not as candidate in the selection criteria at the microarray level [12]. Therefore, we conducted a systematic analysis to evaluate the diagnostic value of miR-21 in detecting breast cancer.

## RESULTS

### Included studies

**Figure 1:**
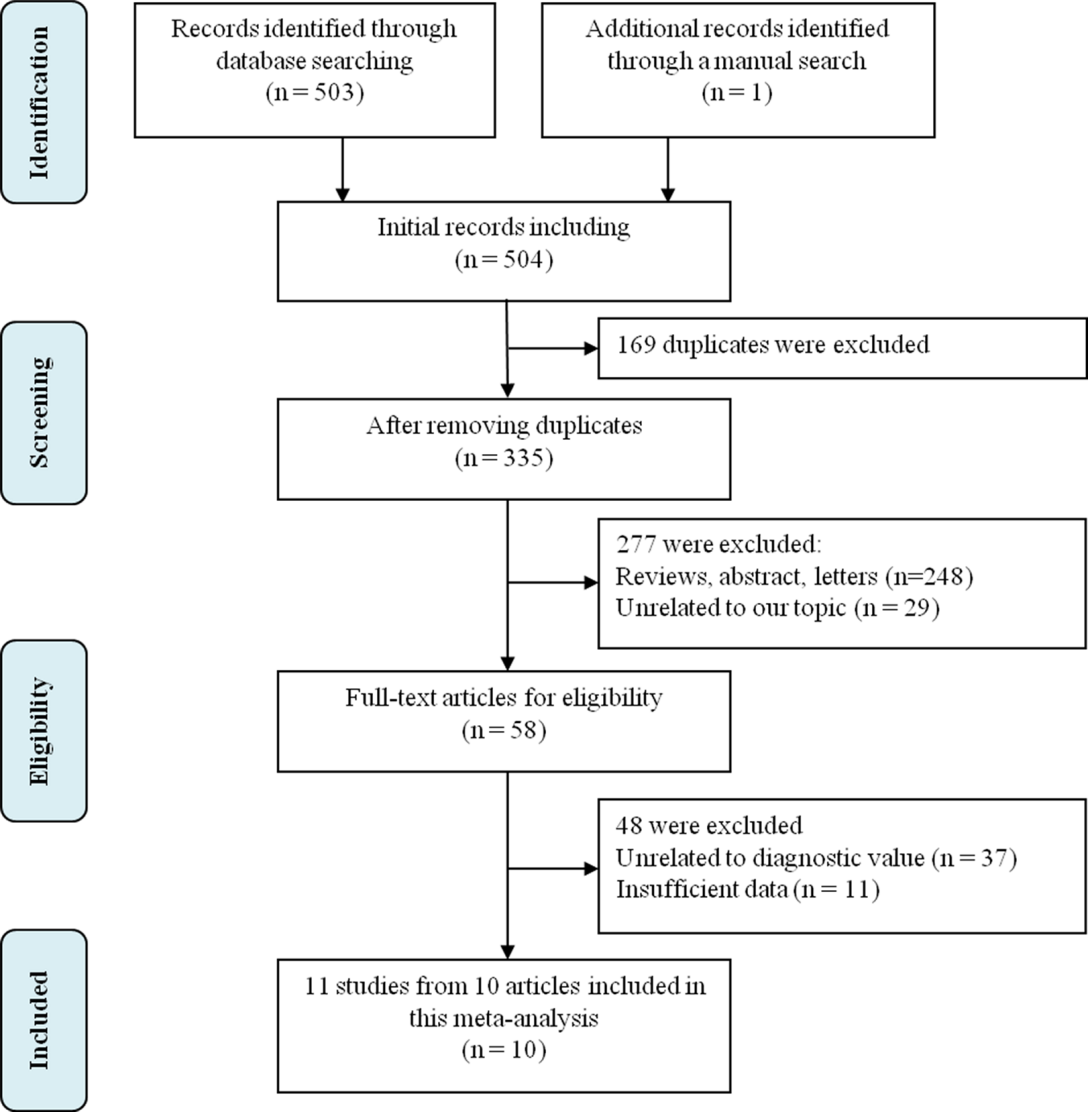
A detailed flowchart of the review process

A detailed flowchart of the review process was presented in Figure 1. A total of 504 articles were identified by initial search, with 503 records identified from database searching and 1 record by manual search. Two independent researchers reviewed articles for duplicates, excluding 169 records. After carefully reviewing titles and abstracts of 335 records, as a result, there were 277. Excluded: 248 were reviews, abstract and letters and 29 were not related to our topic, leaving 58 full-text articles for eligibility. Finally, 11 studies from 10 articles were included in this meta-analysis [10–19].

### Study characteristics and quality assessment

**Figure 2:**
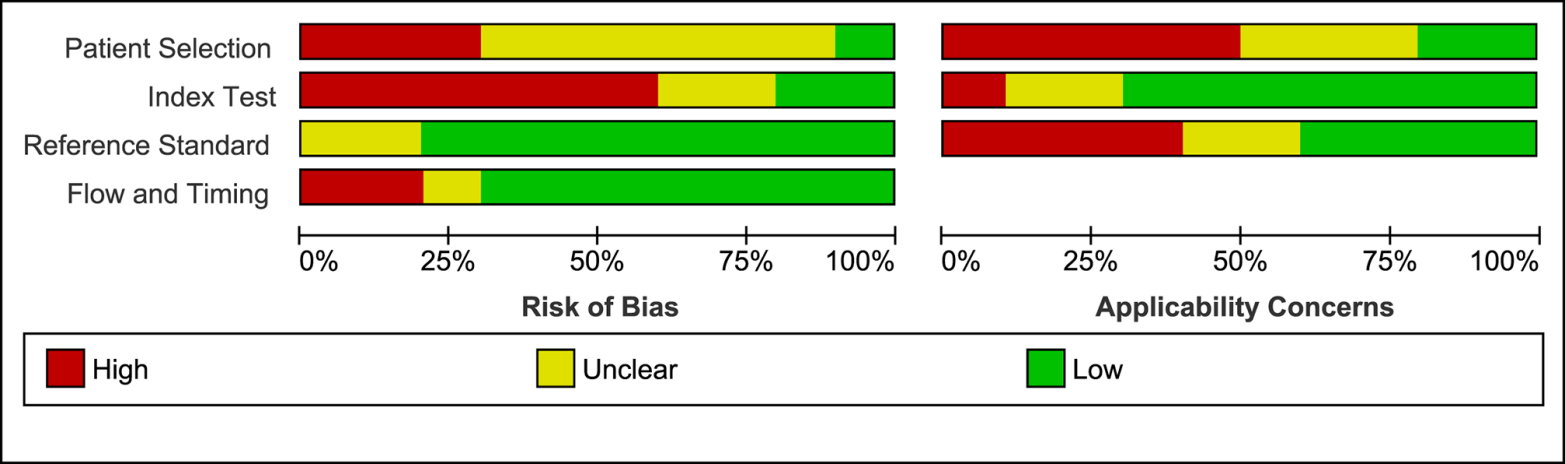
Risk of bias and applicability concerns graph a review of authors' judgments about each domain presented as percentages across included studies

The main characteristics of included studies were summarized in Table 1. Among the 11 studies, 7 studies were conducted in China [10, 11, 13–27], 1 in USA [12], 2 in Mexico [18], and 1 in Egypt [19]. The publication years ranged from 2011 to 2015. A total of 918 breast cancer patients and 613 controls were included. Circulation miR-21 expression levels were measured in serum (*n* = 8), tumor tissue (*n* = 2), and plasma (*n* = 1). In each study, the cutoff values of miR-21 appeared to be different. The quantitative real-time reverse transcription PCR method was used to measure the expression of miR-21. The sufficient data which were used to construct the 2 × 2 table, such as True positive (TP), false positive (FP), false negative (FN), and true negative (TN), were successfully extracted. The quality assessment of the QUADAS-2 tool was shown in Figure 2. Overall, most studies presented they were of high quality relatively.

**Table 1:** Main characteristics of included studies

First author	Year	Country	Ethnicity	Sample size		TP	FP	FN	TN	Cut-off value	Sample types	Reference controls	RNA extraction	Measurements
				Cases	Controls									
Li	2011	China	Asian	33	49	17	3	16	46	18.32	Serum	miR-16	TRIzol	SYBR
Asaga	2011	USA	Caucasian	102	20	72	3	30	17	3.3-dCq	Serum	miR-16	TRIzol	SYBR
Sun	2012	China	Asian	103	55	77	18	26	37	1.358 2^−ΔΔ*Ct*^	Serum	cel-miR-39	Filter cartridge	Taqman
Wang	2012	China	Asian	50	39	40	5	10	34	4.58 2^−ΔΔ*Ct*^	Serum	miR-16	TRIzol	SYBR
Mar-Aguilar	2013	Mexico	Caucasian	61	10	58	2	3	8	6.48 2^−ΔΔ*Ct*^	Serum	18S RNA	miRNAeasy kit	Taqman
Mar-Aguilar	2013	Mexico	Caucasian	50	10	38	4	12	6	6.48 2^−ΔΔ*Ct*^	Tissue	18S RNA	miRNAeasy kit	Taqman
Gao	2013	China	Asian	89	55	78	7	11	48	13.22	Serum	CA153, CEA	TRIzol	SYBR
Lee	2013	China	Asian	110	15	99	4	11	6	2.5 2^−ΔΔ*Ct*^	Tissue	18S RNA	TRIzol	SYBR
Ng	2013	China	Asian	170	100	128	22	42	78	2.34 2^−ΔΔ*Ct*^	Plasma	miR-145	TRIzol	Taqman
Li	2013	China	Asian	120	200	31	46	89	154	NA^*^	Serum	CA153, CEA	Roche Elecsys	Taqman
Toraih	2015	Egypt	Caucasian	30	60	20	8	10	52	7.02 2^−ΔΔ*Ct*^	Serum	RNU6B	Qiagen miRNeasy kit	Taqman

* Data unavailable.

### Diagnostic accuracy and threshold analysis

**Figure 3:**
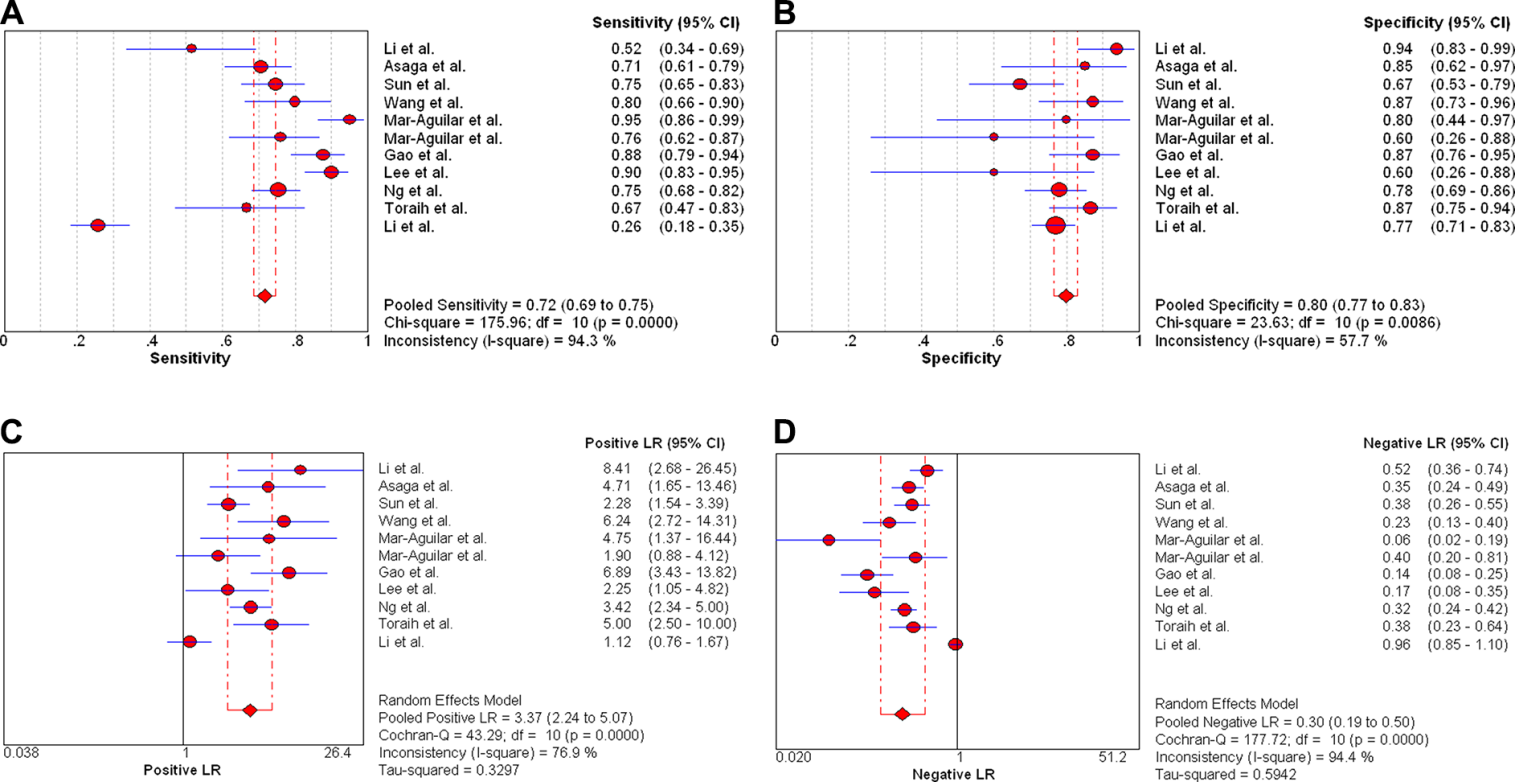
Forest plots of pooled sensitivity (**A**), specificity (**B**), positive likelihood ratio (**C**), and negative likelihood ratio (**D**) for miR-21 in the diagnosis of breast cancer.

**Figure 4:**
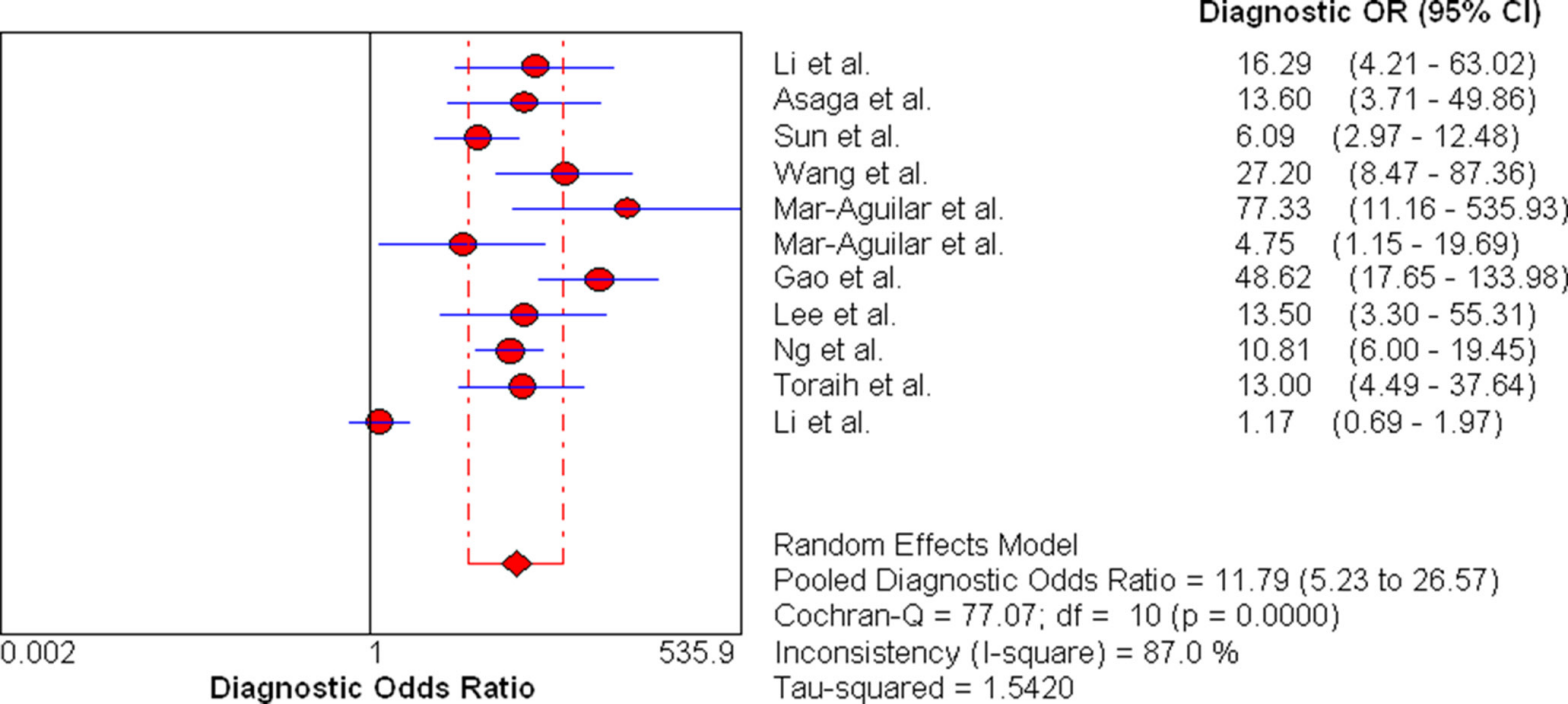
Forest plots of pooled diagnostic odds ratio for miR-21 in the diagnosis of breast cancer

**Figure 5:**
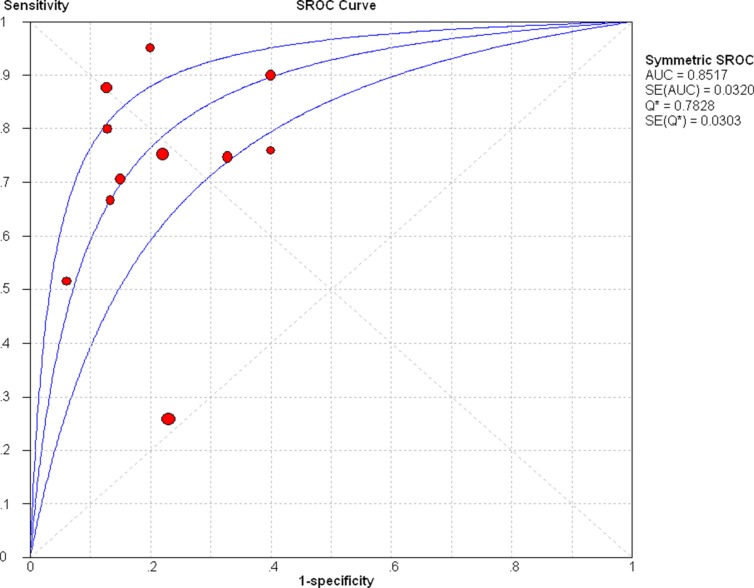
Summary receiver operating characteristic (SROC) curve for miR-21 in the diagnosis of breast cancer

Firstly, we conducted analysis of diagnostic threshold to explore whether the threshold effect was existed in this study, which was an important source of heterogeneity. The results showed that there was no heterogeneity from threshold effect with the spearmen correlation coefficient of sensitivity and 1-specificity of 0.178 (*P* = 0.601). Then Cochran-Q and inconsistency index (*I*^2^) were used to measure whether there was heterogeneity from non-threshold effect in order to choose appropriate calculation model. We used the random effects model to calculate those pooled diagnostic parameters for breast cancer. The pooled sensitivity, specificity, positive likelihood ratio (PLR), negative likelihood ratio (NLR), and diagnostic odds ratio (DOR) with their 95% confidence intervals (CIs) were 0.72 (95% CI: 0.69–0.75, Figure 3A), 0.80 (95% CI: 0.77–0.83, Figure 3B), 3.37 (95% CI: 2.24–5.07, Figure 3C), 0.30 (95% CI: 0.19–0.50, Figure 3D), and 11.79 (95% CI: 5.23–26.57, Figure 4), respectively. The area under the curve (AUC) of SROC was 0.8517 (Figure 5).

### Meta-regression, subgroup analysis and publication bias

We also performed meta-regression analysis to explore source of heterogeneity based on ethnicity, sample size (≥ 100 vs. < 100), sample source, reference controls, RNA extraction, measurements (Table 2). The results showed that none of the above covariates contributed the heterogeneity (all *P* > 0.05). Then we conducted subgroup analysis based on those covariates. The results of different subgroups were relatively consistent with the major results, which suggested that our results were relatively credible (Table 3). Moreover, Egger' test (*P* = 0.909) or Begg's test (*P* = 0.488) was detected and the results showed that there was no significant publication bias in our study.

**Table 2:** Results of the multivariable meta-regression model for the characteristics with backward regression analysis (Inverse variance weighs)

Variables	Coefficient	Standard Error	*P*	RDOR	95% CI
Cte	5.451	0.7895	0.0023	−--	−--
S	0.205	0.1766	0.3095	−--	−--
Ethnic	0.424	0.4500	0.3992	1.53	0.44–5.33
Sample size	−1.304	0.5388	0.0519	0.27	0.08–1.04
Sample types	−0.243	0.2440	0.3568	0.78	0.43–1.42
Reference controls	−0.056	0.2244	0.8178	0.95	0.46–1.93
RNA extraction	−1.131	0.5786	0.0863	0.32	0.08–1.23
Measurements	2.768	1.2297	0.0742	15.92	0.67–375.70

**Table 3:** Results of subgroup analysis in diagnostic accuracy of miR-21 for breast cancer

Subgroup	No. of studies (No. of cases)	Sensitivity (95% CI)	Specificity (95% CI)	PLR (95% CI)	NLR (95% CI)	DOR (95% CI)	AUC
Ethnicity							
Asian	7 (675)	0.70 (0.66–0.73)	0.79 (0.76–0.83)	3.27 (1.92–5.56)	0.33 (0.17–0.62)	10.64 (3.66–30.97)	0.8472
Caucasian	4 (243)	0.77 (0.72–0.82)	0.83 (0.74–0.90)	3.65 (2.18–6.11)	0.29 (0.17–0.49)	13.73 (5.54–34.04)	0.8625
Sample size							
≥ 100	5 (605)	0.67 (0.63–0.71)	0.76 (0.71–0.80)	2.32 (1.41–3.82)	0.38 (0.19–0.75)	6.31 (2.09–19.00)	0.7935
< 100	6 (313)	0.80 (0.75–0.84)	0.87 (0.82–0.91)	4.89 (3.13–7.62)	0.26 (0.15–0.44)	20.89 (10.14–43.06)	0.8948
Sample types							
Serum	8 (588)	0.67 (0.63–0.71)	0.81 (0.77–0.85)	3.95 (2.19–7.12)	0.31 (0.17–0.57)	13.46 (4.37–41.41)	0.8865
Tissue	2 (160)	0.86 (0.79–0.91)	0.60 (0.36–0.81)	2.07 (1.20–3.56)	0.26 (0.11–0.62)	8.04 (2.86–22.58)	—
Plasma	1 (170)	—	—	—	—	—	—
Reference control							
miR-16	3 (185)	0.70 (0.63–0.76)	0.90 (0.83–0.95)	6.18 (3.51–10.89)	0.36 (0.24–0.55)	18.81 (9.06–39.06)	0.8954
18S RNA	3 (221)	0.88 (0.83–0.92)	0.67 (0.47–0.83)	2.37 (1.44–3.89)	0.17 (0.06–0.46)	15.09 (3.49–65.19)	0.5981
CA153, CEA	2 (209)	0.82 (0.74–0.89)	0.87 (0.79–0.93)	5.86 (3.59–9.58)	0.23 (0.09–0.63)	15.40 (6.97–92.54)	—
RNA extraction							
TRIzol	6 (554)	0.78 (0.75–0.82)	0.84 (0.79–0.88)	4.45 (3.02–6.54)	0.28 (0.19–0.40)	17.90 (10.63–30.15)	0.8800
Others	5 (364)	0.62 (0.56–0.67)	0.77 (0.72–0.81)	2.36 (1.35–4.13)	0.36 (0.17–0.77)	6.84 (1.95–23.97)	0.8058
Measurements							
SYBR	5 (384)	0.80 (0.75–0.84)	0.87 (0.81–0.92)	5.02 (3.09–8.16)	0.26 (0.16–0.43)	23.59 (13.66–40.73)	0.8974
Taqman	6 (534)	0.78 (0.73–0.81)	0.77 (0.71–0.82)	3.02 (2.17–4.19)	0.32 (0.22–0.45)	10.03 (5.50–18.26)	0.8289

## DISCUSSION

We performed a systematic review to evaluate the diagnostic value of miR-21 as a potential diagnostic biomarker for breast cancer patients. Our finding suggested that the pooled sensitivity, specificity, PLR, NLR and DOR were 0.72 (95% CI: 0.69–0.75), 0.80 (95% CI: 0.77–0.83), 3.37 (95% CI: 2.24–5.07), 0.30 (95% CI: 0.19–0.50) and 11.79 (95%CI: 5.23–26.57), respectively. The AUC of SROC was 0.8517.

Currently, a number of convenient and novel biomarkers have been established in the routine evaluation of breast cancer. Although estrogen receptor (ER) and human epidermal growth factor receptor-2 (HER2) for predicting the response to endocrine and biological therapies are already available, their performances are far from perfect. For example, there were still some non-responding patients in the assessment of ER and HER2 status [20, 21]. In addition, other molecular biomarkers, such as CEA, cytokeratin fragment (CYFRA 21-1), and neuron specific enolase (NSE), were limited in the clinic with their low sensitivity and specificity [22].

Recently, various studies showed that abnormal expression of miRNAs played an important role in the pathogenesis, metastasis and prognosis for breast cancer [23, 24]. Some studies reported that miR-21 might be as a potential biomarker for breast cancer diagnosis because breast cancer patients had higher serum miR-21 expression than healthy women [25, 26]. In our meta-analysis, the pooled sensitivity and specificity were 0.72 and 0.80, which indicated that the diagnostic accuracy may not be high enough as expected. The results were consistent with the recently published studies by Li et al. and Shen et al. [27, 28]. However, compared with some traditional biomarkers, such as CEA, NSE (with sensitivities of 0.48 and 0.39), miR-21 still had higher diagnostic value in detecting breast cancer. The PLR and NLR were used to estimate the diagnostic accuracy in clinical level. The pooled PLR of 3.37 suggested that breast cancer patients could have about 3.37-fold higher chance of being miR-21 positive compared to healthy controls. The pooled NLR of 0.30 indicated that the possibility of individuals having cancer was 30% if the miR-21 was negative. Moreover, the value of DOR ranged from 0 to infinity, with higher value meaning better test discrimination [29]. The area under curve is another parameter to evaluate the diagnostic value. The ideal SROC curve position is near the upper-left corner which would imply a perfect test [30]. Statistically, if the range of AUC was 0.97 or above which was considered to have excellent accuracy; the range of AUC 0.93–0.96 was considered to be very good; the range of AUC 0.75–0.92 was considered to be good; and a range of AUC less than 0.75 should be cautiously to evaluate the accuracy which might be a random test [31]. Our results of DOR and AUC was 11.79 and 0.8517, respectively, which indicated the overall accuracy in diagnosing breast cancer was high.

Exploring the sources of heterogeneity is important in a meta-analysis. In this study, there was no heterogeneity from threshold effect with the spearmen correlation coefficient of sensitivity and 1-specificity of 0.178. However, substantial heterogeneity was found during the analyzing several parameters. Meta-regression and some subgroup analyses were conducted according to the majors attributes of primary studies. In present study, different measures such as reference controls, RNA extraction and measurement methods, were used to extract miR-21 in different studies. All these variables may influence the heterogeneity. As a result, we failed to find the sources. On the other hand, we also analyzed miR-21 diagnostic efficiency in three different sample types. The subgroup analysis showed that serum-based miR-21 had higher accuracy than miR-21 in tissue for diagnosing breast cancer. Other results of different subgroups were relatively consistent with the major results, which suggested that our results were relatively credible.

There were several potential limitations in our study. Firstly, the results may suffer from publication bias in our study, because studies with null results tend not to be published. Publication languages were limited to English and Chinese. Other potentially eligible studies which met our inclusion criteria may not be included. Secondly, sample sizes of studies included in this meta-analysis were small, which may appear a small-study effect. Thirdly, only Asian and Caucasian populations were considered in subgroup analysis, with no African population involved, which may cause selection bias from population.

In conclusion, our analyses suggested that miR-21 is a promising biomarker in diagnosing breast cancer. For clinical purpose, further large-scale studies are warranted to validate its clinical application.

## MATERIALS AND METHODS

Our present study was performed in accordance with the guidelines for the Preferred Reporting Items for Systematic reviews and Meta-Analyses (PRISMA) [31].

### Search strategy

Two researchers independently searched PubMed, Embase, Chinese National Knowledge Infrastructure (CNKI), Wan Fang Data, and VIP database to identify relevant studies which evaluated the diagnostic value of miR-21 for breast cancer patients, up to November 9, 2015. A manual review of relevant publications was also performed to obtain additional studies. The following search terms were used to retrieve articles and abstracts: (microRNA-21 or micro RNA 21 or miRNA-21 or miR-21) and (breast cancer or breast tumor or breast neoplasm or breast carcinoma). Only the most recent or the largest sample size study was included in the final analysis. Publication languages were limited to English and Chinese.

### Study selection

Studies included in present meta-analyses should meet the following criteria: (1) diagnostic effect about miR-21 for breast cancer; (2) breast cancer was confirmed by pathological examination; (3) the levels of miR-21 in tissue or serum was determined; (4) sensitivity, specificity, and cut-off values can be found in identified studies or calculated from the provided data. While the exclusion criteria were listed as follow: (1) studies without sufficient data to construct the 2 × 2 table; (2) Meta-analyses, reviews, comments, letters, editorial articles, conference abstracts, meeting, and animal and cell studies; (3) publications were identified as duplicates.

### Data extraction

Two researchers reviewed the abstract first independently and then summarized the full selected articles. Any disagreements were resolved by discussion or consulting the third reviewer. The relevant data were extracted as follow: first author, publication years, country of origin, ethnicity, number of patients and controls, true and false positive and negative, cut-off value, sample types, reference control, RNA extraction, measurements.

### Quality assessment

Quality Assessment of Diagnostic Accuracy Studies 2 (QUADAS-2) [32] was used to assess the quality of the studies included in this meta-analysis independently by the same two researchers. Each of the assessment has seven questions with the answered with “yes”, “no”, or “unclear”. The answer of “yes” means that a study's risk bias can be judged as low, while “no” and “unclear” mean that the risk of bias can be judged as high.

### Statistical analysis

Pooled sensitivity, pooled specificity, positive likelihood ratio, negative likelihood ratio, diagnostic odds ratio, and corresponding 95% CIs were calculated to evaluate the diagnostic value of miR-21. Summary receiver operator characteristics which shows the relationship between sensitivity and 1-specificity, was used to evaluate the consistency of results among all studies and the accuracy of the diagnostic test. The Spearman correlation coefficient was used to test the diagnostic threshold effect, which may produce significant heterogeneity (*P* < 0.05). Additionally, the chi-square, *Q* value and *I*^2^ test were used to assess the heterogeneity from non-threshold effect. A value of *P* less than 0.1 or an *I*^2^ ≥ 50% indicated the existence of significant heterogeneity. Meta-regression and subgroup analyses were conducted to explore sources of heterogeneity. Egger's test [33] and Begg's test [34] were performed to examine the potential publication bias. All statistical analyses were performed using Meta-Disc 1.4 and Stata 12.0 [35].
